# A Natural Impact: NK Cells at the Intersection of Cancer and HIV Disease

**DOI:** 10.3389/fimmu.2019.01850

**Published:** 2019-08-14

**Authors:** Olivier Lucar, R. Keith Reeves, Stephanie Jost

**Affiliations:** ^1^Center for Virology and Vaccine Research, Beth Israel Deaconess Medical Center, Harvard Medical School, Boston, MA, United States; ^2^Ragon Institute of Massachusetts General Hospital, MIT, and Harvard, Cambridge, MA, United States

**Keywords:** HIV, cancer, natural killer, innate immunity, immunotherapy

## Abstract

Despite efficient suppression of plasma viremia in people living with HIV (PLWH) on cART, evidence of HIV-induced immunosuppression remains, and normally benign and opportunistic pathogens become major sources of co-morbidities, including virus-induced cancers. In fact, cancer remains a primary cause of death even in virally suppressed PLWH. Natural killer (NK) cells provide rapid early responses to HIV infection, contribute substantially to disease modulation and vaccine protection, and are also major therapeutic targets for cancer immunotherapy. However, much like other lymphocyte populations, recent burgeoning evidence suggests that in chronic conditions like HIV, NK cells can become functionally exhausted with impaired cytotoxic function, altered cytokine production and impaired antibody-dependent cell-mediated cytotoxicity. Recent work suggests functional anergy is likely due to low-level ongoing virus replication, increased inflammatory cytokines, or increased presence of MHC^low^ target cells. Indeed, HIV-induced loss of NK cell-mediated control of lytic EBV infection has been specifically shown to cause lymphoma and also increases replication of CMV. In this review, we will discuss current understanding of NK cell modulation of HIV disease, reciprocal exhaustion of NK cells, and how this may impact increased cancer incidences and prospects for NK cell-targeted immunotherapies. Finally, we will review the most recent evidence supporting adaptive functions of NK cells and highlight the potential of adaptive NK cells for cancer immunotherapy.

## NK Cells Have Therapeutic Potential to Enhance Control of Both HIV and HIV-Related Cancers

While efficient suppression of plasma viremia by combination antiretroviral therapy (cART) has substantially decreased mortality of people living with HIV (PLWH), burgeoning evidence suggests a higher occurrence of a vast range of comorbidities linked to long-term treatment and aging among PLWH, including cancers. The incidence of AIDS-defining cancers such as Kaposi Sarcoma, Non-Hodgkin lymphoma, and cervical cancer, has substantially decreased with access to cART. However, cART-treated PLWH still have a higher susceptibility to non-AIDS defining cancers (NADCs) compared to the general population, and NADCs currently represent a major cause of mortality among PLWH (1, 2). In particular, lymphomas, including Burkitt and classical Hodgkin lymphomas, have been reported at a significantly higher frequency in PLWH, yet many other cancers associated with infections (i.e., anus, oropharynx, liver) and some cancers associated with cigarette smoking (i.e., lung, kidney) were also found to be elevated among PLWH (3). Several mechanisms have been proposed to explain the predisposition of cART-treated PLWH to NADCs (4). Nevertheless, as cART treatment only partially prevents HIV-induced chronic inflammation and immune senescence, it is very likely that immune dysregulation in PLWH is an important determinant of NADCs and explains why most cancers predominantly found in PLWH are related to viral infections (4, 5).

### NK Cell Subpopulations

Natural killer (NK) cells are large granular leukocytes that play a central role in the control of viral infections and neoplasms. Human NK cells are defined as CD3^neg^CD56^pos^ lymphocytes (6) and can be subdivided into functionally distinct subpopulations based on expression levels of CD56 and CD16 (7). CD56^bright^CD16^neg^ NK cells have a high proliferation potential and the ability to secrete a large amount of cytokines, notably IFN-γ in response to IL-12, with limited cytotoxic functions (8), while CD56^dim^CD16^pos^ NK cells display strong cytolytic activity as well as a significant capacity to secrete cytokines upon triggering of activating receptors (6, 9). In addition, a subset of CD56^neg^CD16^pos^ NK cells appears to expand in chronic viral infections including HIV and might represent an exhausted/anergic subset of NK cells (10–12).

Our understanding of human NK cells has essentially been acquired while studying peripheral blood NK cells, yet it is now clear that subsets other than CD56^bright^ and CD56^dim^ NK cell subpopulations can be found in peripheral tissues. Tissue-resident NK cells differ from circulating NK cells and are found not only in secondary lymphoid organs but also in many peripheral tissues including the uterus, lung, and liver where they represent up to 50% of lymphocytes (13–15). Findings from recent studies have allowed reliable identification of tissue-resident NK cells based on their expression of CD69, CD49a, or CD103, three markers functionally involved in the retention of lymphocytes in tissues. Besides the uterus, lung and liver, NK cells have been characterized in many additional tissues such as the intestinal mucosa, skin, and kidneys. However, in a majority of older studies it is not clear if those NK cells represent tissue-resident NK cells, NK cells circulating between tissues and blood, or innate lymphoid cells (ILCs). Indeed, ILCs can express markers associated with NK cells such as CD56, NKp46, or NKp44, and it was only lately appreciated that a deeper analysis of expressed transcription factors and produced cytokines is required to discriminate NK cells and ILCs. Until recently, NK cells were even considered as part of ILC group 1 due to the common innate lymphoid progenitors. However, NK cells are now distinguished from other ILCs because of their unique development and cytotoxic functions (16). In summary, tissue-resident NK cells likely play a crucial role in select tissues or organs involved in cancer and HIV disease, yet due to the scarcity of data on the contribution of tissue-resident NK cells in HIV infection or cancer development, herein we will focus primarily on circulating NK cells.

### NK Cell Function

NK cells can efficiently discriminate between transformed or virally-infected cells and normal cells without the need for prior sensitization, and have the capacity to kill abnormal cells before adaptive immunity develops, thereby containing viral replication or tumor development. NK cells can clear cellular targets by a number of different mechanisms, including (i) exocytosis of cytotoxic granules containing perforin and granzyme that results in cell lysis, (ii) signaling through Fas ligand or TRAIL death receptors which induces apoptosis, (iii) release of cytokines with potent anti-viral and anti-tumor activities, and (iv) antibody-dependent cellular cytotoxicity (ADCC), triggered through binding of the FcγRIIIA receptor (CD16) on NK cells by the constant (Fc) domain of IgG antibodies. NK cells also play major roles in tuning and controlling adaptive immune responses (17).

### NK Cell Receptors

Unlike other lymphocytes, NK cells lack antigen-specific receptors but lyse target cells following the integration of inhibitory and activating signals. These signals are generated by an arsenal of germline encoded cell surface molecules, with effector functions taking place when activating signals overcome inhibitory ones (18). The major NK cell receptors, which allow NK cells to discriminate between “self” and a variety of pathological cell states belong to three main categories: (i) natural cytotoxicity receptors (NCRs) such as NKp46, NKp30, and NKp44, which can bind to several viral or tumor-associated molecules (19, 20), (ii) NKG2A/C/E-CD94 heterodimers and NKG2D homodimers, which are c-type lectins binding to the non-classical Human Leukocyte Antigen E (HLA-E) molecule and stress-induced ligands, respectively, and (iii) the killer-cell immunoglobulin-like receptors (KIRs), which primarily recognize HLA class Ia (HLA-Ia) and Ib (HLA-Ib) molecules and related surface molecules (21).

The classical HLA-Ia group includes the highly polymorphic and ubiquitously expressed HLA-A, -B and -C antigens. Non-classical HLA-Ib antigens comprise HLA-E, -F, and -G molecules which are expressed in a tissue-specific manner, display low genetic diversity, and limited peptide repertoire (22). While the biological function and clinical relevance of most HLA-Ia and -Ib antigens have been investigated in detail, HLA-F was only recently recognized for its important immune-regulatory functions in cancer (23–26) and potentially in HIV infection (27). Besides their role in mediating recognition and elimination of unhealthy cells, a direct interaction between inhibitory KIRs and their HLA class I ligands during NK cell development is necessary for NK cells to acquire self-tolerance and functionality through an education process termed “licensing.” Besides NK cell licensing, which involves engagement of self-HLA class Ia molecules by their inhibitory ligand, non-classical HLA class I as well as non-HLA class I molecules also contribute to NK cell education (28).

While NKp30, NKp46, NKG2D, and NKG2C are expressed at relatively comparable levels on circulating CD56^dim^ and CD56^bright^ NK cells, other major NK cell receptors are differentially expressed on distinct subsets of NK cells (11). Peripheral blood CD56^bright^ NK cells have been proposed to represent a mixture of immature NK cells that are direct precursors of CD56^dim^ NK cells (29, 30), and mature NK cells, including CD56^dim^ NK cells that have upregulated CD56 and lost CD16 upon activation in peripheral tissues (31). Immature CD56^bright^ NK cells lack expression of KIRs, which are sequentially acquired during the differentiation process into mature CD56^dim^ NK cells, a process that occurs in parallel with a progressive decrease in NKG2A expression and acquisition of the marker of terminal differentiation CD57 (32). NKp44 is usually not expressed on peripheral blood NK cells and up-regulated upon IL-2- or IL-15-mediated NK cell activation (33).

Other groups of receptors have received attention because their expression on NK cells is modulated in HIV and/or cancer and impacts NK cell function. These include Signaling Lymphocyte Activation Molecule (SLAM)-related receptors such as 2B4 (34–37) that displays co-stimulatory functions on NK cells and binds to CD48, or sialic acid-binding immunoglobulin-type lectins (Siglec), which are HLA class I-independent inhibitory receptors that recognize sialic acid-containing carbohydrates (38, 39). T cell immunoglobulin and mucin-domain containing-3 (Tim-3), which can binds to galectin-9, carcinoembryonic antigen cell adhesion molecule 1 (Ceacam1), high-mobility group box 1 (HMGB1) or phosphatidylserine (PtdSer), is another immunoregulatory molecule highly expressed on NK cells with relevance for NK cell function in both HIV and cancer (40–47). NK cells also express members of the immunoglobulin (Ig) superfamily such as the activating receptor DNAM-I (48–51), which has been shown to recognize CD112 (PVR) and CD155 (Nectin-2), two ligands expressed on tumor cells.

### NK Cell Control of Cancers and HIV Infection

NK cells were originally defined as immune cells capable of lysing tumor cell lines. Since then, their capacity to kill primary cancer cells *in vitro* as well as their ability to prevent growth and metastasis of certain tumors *in vivo*, principally hematological cancers, has been clearly established (52–54). In particular, protection against development of cancer has been associated with higher NK cell cytotoxicity (55) and increasing evidence has highlighted the implication of NK cells in defense against leukemia. Importantly, in the context of hematopoietic stem cell transplantation (HSCT), it has been demonstrated that allogeneic NK cells from the donor can prevent relapse of myeloid leukemia via graft-vs.-leukemia effect (56, 57). However, thus far clinical trials aimed at harnessing NK cell anti-tumor activity have shown marginal therapeutic efficacy (58–61), with beneficial effects reported mainly against hematologic malignancies (62). Development of therapeutic strategies to enhance NK cell activity against tumor cells *in vivo* has therefore become a major field of investigation.

Besides NK cell anti-metastatic properties, numerous studies have emphasized the early and pivotal role of NK cells in the control of HIV infection. Notably, particular KIR genes expressed in conjunction with their HLA ligands are associated with significantly slower HIV disease progression and lower viral set-point (63, 64), elite control of HIV (65), and protection against disease acquisition (66, 67). In particular, activating KIR3DS1 has been associated with delayed HIV disease progression in individuals with specific HLA-B alleles since a first study by Martin et al. (63), yet a ligand for KIR3DS1 was only recently described, underscoring the relevance of HLA-F in regulating immunity to HIV (27). Indeed, HLA-F open conformers (OCs), which constitute heavy chains not bound to β_2_-microglobulin, can be recognized by several KIRs but have the highest affinity for KIR3DS1 (27, 68). HLA-F OCs trigger polyfunctional responses by KIR3DS1^pos^ NK cells, which efficiently suppress HIV replication *in vitro*. HLA-F is expressed on activated CD4^pos^ T cells and may act as a marker of cellular stress in specific conditions including viral infections and cellular transformation.

Control of HIV infection has also been associated with NK cells displaying potent cytotoxic function and IFN-γ expression after stimulation (69) as well as with polyfunctional CD8α^pos^ NK cells (70). Moreover, it has been demonstrated that NK cells expand in the peripheral blood during early acute HIV infection, can inhibit HIV replication *in vitro*, and can mediate *in vivo* immune pressure in infected individuals, resulting in viral escape (71–77). Finally, indirect NK cell-mediated ADCC has been linked to vaccine-induced protective immunity against HIV infection (78), elite control of HIV (79–81) and slower HIV disease progression (82, 83). Therefore, in cART-treated PLWH, therapeutic interventions targeting NK cells might result in improved control of HIV and other viral infections as well as in decreased incidence of cancers.

## Aberrant Expression of Key NK Cell Receptors May Contribute to Decreased Control of Pre-cancerous Cells in PLWH

NK cell-mediated immunosurveillance is decreased in PLWH, mostly as a long-term consequence of chronic HIV infection. While administration of suppressive cART partly restores NK cell properties, NK cells undergo many HIV-associated functional and phenotypic alterations, which are likely to severely impair NK cell-mediated control of viruses as well as of pre-cancerous cells.

**Figure 1:**
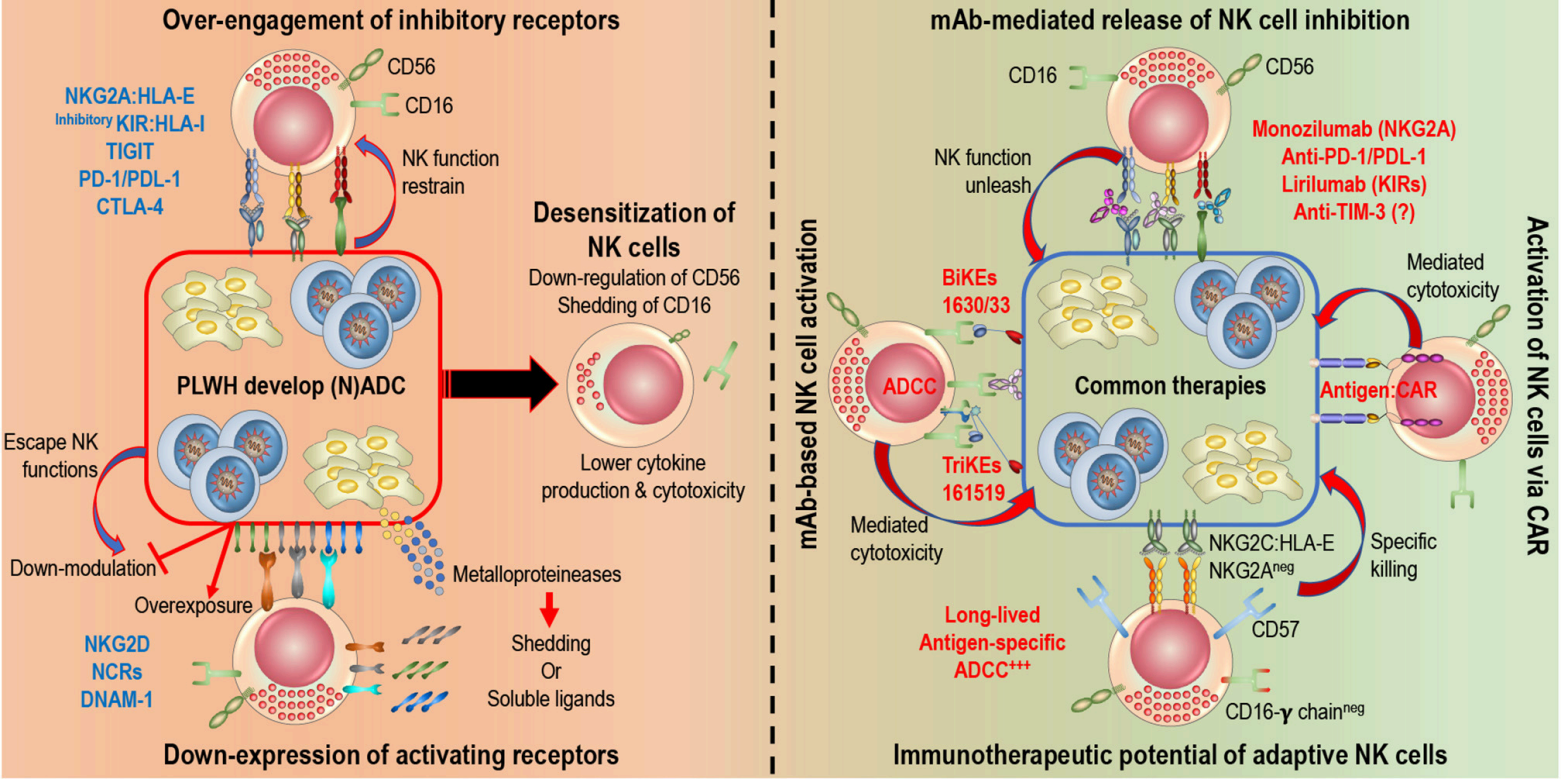
Rescuing and harnessing NK cell potency in PLWH developing cancers. **Left**: HIV-infected and cancer cells share common NK cell escape mechanisms. 1. Over-engagement of inhibitory receptors (i.e., NKG2A, inhibitory KIRs, PD-1…) blocks killing abilities of NK cells. 2. Down-modulation (blocking expression of ligand or shedding of ligand) or over-exposure (constant expression of ligands or release of soluble ligands) induce down-expression of activating receptors (NKG2D, NCRs, DNAM-1…) on NK cells. **Right**: Novel immunotherapies are being develop to harness NK cell potency and target HIV-infected and cancer cells. 1. Monoclonal antibodies (mAb) release engagement of inhibitory receptors, unleash NK cell cytotoxicity and engage Fc receptors (CD16) to induce ADCC. Several clinical trials are in progress. 2. Engineered proteins, Bi-specific or Tri-specific Killer engagers (BiKEs or TriKEs) and Chimeric Antigen Receptors (CARs), act as a link between NK cells and target cells to induce cytotoxicity. BiKEs or TriKEs induce ADCC by engaging CD16 receptors and bind to antigen on target cells. 3. Adaptive features of NK cells, defined by a higher expression of CD57, NKG2C, and/or absence of FcR-γ, could be harnessed to elicit specific killing of target cells.

Engagement of the well-described NCRs, NKG2D, and CD16 receptors represent major pathways to promote potent NK cell activation and cytotoxic responses. In both chronic HIV infection and cancer, NK cell recognition of abnormal cells through those activating receptors is defective, mainly as a result of chronic exposure to the respective ligands, which results in persistent down-modulation of NCRs, NKG2D, and CD16 on NK cells. In this section, we will review known effects that malignancies and HIV infection have on the expression of key NK cell receptors (Figure 1, left panel). It is important to note that a simplified definition of NK cells as CD3^neg^CD56^pos^ lymphocytes or different gating strategies to identify the major NK cell subsets represent a caveat of some older studies, precluding any definite conclusions on phenotypic alterations specifically affecting individual NK cell subsets.

NCRs represent a particularly important family of activating receptors in NK cell-mediated elimination of tumor cells, with a few tumor-associated ligands described for those molecules thus far (19). Accordingly, strategies to escape immune recognition by NCRs have been reported in both HIV infection and cancer, and have been associated with dysfunctional NK cells expressing lower levels of NCRs than NK cells from control subjects in many studies. Upon HIV infection, a population of dysfunctional CD56^neg^CD16^pos^ NK cells expands at the expense of the CD56^dim^CD16^pos^ NK cell subset and is progressively eliminated with cART treatment. In PLWH, decreased NK cell expression of NKp30 and NKp46 receptors has been reported, and appears to be a characteristic of CD56^neg^CD16^pos^ NK cells, reducing their cytokine production and cytotoxicity, notably against tumor target cells, as well as their ability to interact with other immune cells (84–86). Similarly, decreased NK cell cytotoxicity in patients with acute or chronic myeloid leukemia (AML or CML) correlates with lower levels of NKp30 and NKp46 expression on NK cells compared to healthy individuals (87–89). NCRs downregulation on NK cells is induced by cell-to-cell contact with AML blasts and linked to poor survival in AML patients (87), whereas high levels of NKp30 and NKp46 expression on NK cells at AML diagnostic are predictive of better outcomes (90, 91). In AML, high expression of the immunosuppressive glycoprotein CD200 on tumor cells has been shown to directly impair NK cell anti-tumor responses and is associated with downregulated expression of NKp44 and NKp46 receptors on NK cells (92).

As overexposure to their ligands promotes decreased NCRs expression on NK cells, it is not surprising that shedding of NCR ligands is a hallmark of tumor escape, underscoring further the importance of this family of receptors in anti-metastatic NK cell functions. The A disintegrin and metalloproteinase ADAM-10 and ADAM-17 can cleave B7–H6, a ligand for NKp30, from the surface of tumors, likely leading to reduced NKp30 expression on NK cells surrounding the tumor (93, 94). Shedding of NKp30 ligands has also been described in chronic lymphocytic leukemia (CLL), in which exosomal expression of BAG6 mediates NK cell activation, whereas soluble BAG6 suppresses NK cell cytotoxicity (95). Galectin-3 is another molecule released by tumor cells that can serve as ligand for NKp30 and prevent NK cell activation (96). As another immune escape mechanism, catabolites specifically generated in tumor microenvironments, such as L-kynurenine, can also directly down-modulate NKp46 expression on NK cells (97). Whether HIV infection-associated NCR ligands are shed from the surface of infected cells remains to be fully determined, but likely contributes to impaired NCR^pos^ NK cell function in HIV infection.

Altogether, these data suggest that fully restoring and even enhancing NCR-mediated signaling in NK cells might be crucial to efficiently control pre-cancerous cells in PLWH. Of note, B7–H6 is the only NCR ligand expressed on tumors that has been characterized so far. Identification of NCR ligands specifically expressed in cancer or HIV infection would represent a milestone in the development of therapeutic interventions aimed at maintaining NCR-mediated NK cell function in PLWH. Finally, given the crucial role played by NCRs in regulating NK cell function in both blood and tissues, therapeutic interventions to enhance tumor surveillance by NK cells and targeting NCR signaling are currently being explored (20).

NKG2D is one of the most important NK cell activating receptor in terms of recognition and elimination of abnormal cells expressing stress-induced ligands. Similarly to NCRs, tumors and HIV evolved immune escape mechanisms to specifically circumvent NKG2D-mediated recognition by NK cells. In HIV infection, reduced NKG2D expression on NK cells and dampened NK cell function have been linked to elevated levels of the soluble form of its major histocompatibility complex I-related chains A (MICA) ligand in patient sera (98). MICA is likely released by HIV-infected CD4^pos^ T cells based on their increased expression levels of matrix metalloproteinases MMP-2 and -7, a family of enzymes previously described for their role in proteolytic shedding of NKG2D ligands in human tumors (99, 100). UL16 binding proteins (ULBP) also serve as ligands for NKG2D and their expression is induced on HIV-infected cells (34), yet levels of ULBP-1 and -2 is down-modulated by the HIV accessory protein Nef, thereby dampening NKG2D-mediated NK cell cytotoxicity (101).

Tumor progression has been associated with lower levels of NKG2D (as well as NKp30 and NKp46) expression on NK cells from patients with cervical cancer (102), and defective NK cell function owing to NKG2D downregulation has been linked to high-risk myelodysplastic syndrome (MDS) (103). Shedding of NKG2D ligands also plays a central role in tumor escape. In AML patients, chronic exposure to MICA/B decreases expression of NKG2D on NK cells (104) and the concentration of NKG2D soluble ligands in the peripheral blood correlates with reduced NK cell cytotoxicity in AML and CML (105). MICA is released in multiple myeloma (106, 107), and MICA/B as well as ULBP-6 are shed from leukemic cells (108). NKG2D ligand shedding is also involved in Hodgkin lymphoma, in which lymph node stromal cells express proteases that shed MICA and ULBP-3 from the surface of the lymphoma cells (109). Thus, NKG2D and its well-described ligands represent additional promising therapeutic target to enhance immunosurveillance by NK cells in PLWH. Accordingly, it has been recently demonstrated that antitumor responses by NK cells can be efficiently promoted by antibodies against MICA by blocking MICA/B shedding and coating MICA-expressing tumor cells, rendering them susceptible to ADCC (110).

Function of additional NK cell receptors is modulated by HIV infection and play an important role in NK cell responses to cancerous cells, including the activating receptor DNAM-1 that is expressed on the majority of peripheral blood NK cells (111–115). The CD155 ligand for DNAM-I has been shown to be present on HIV-infected T cells and, although discrepant results were obtained based on the cell culture model used, some studies found CD155 to be counter-regulated by the HIV proteins Nef and Vpu, thereby preventing NK cell activation (50, 51, 116). Many tumors also express ligands for DNAM-1, triggering NK cell cytokine production and cytotoxicity (117, 118). Tumor escape from DNAM-1 has been described and associated with DNAM-1 downregulation on NK cells isolated from patients with cancer (119–124).

Siglec receptors, and particularly Siglec-7 and -9, have also gained a lot of attention in the past decade for their involvement in immune evasion of tumor and virus-infected cells. Siglec-7 and Siglec-9 are constitutively expressed on all peripheral blood NK cells and on a mature subset of cytotoxic CD56^dim^ NK cells, respectively (125). Reduced Siglec-7 expression marks a subset of dysfunctional NK cells that appears in early stages of HIV infection, prior to downmodulation of CD56, in subjects with elevated HIV replication, and also characterizes the dysfunctional CD56^neg^ NK cell subset in chronic HIV infection (126, 127). Interestingly, an association between downregulation of Siglec-7 and dysfunction of NK cells has also been described in HIV-2 infection (128).

Siglec-7 and -9 ligands are widely expressed on distinct tumor cells and shield them from Siglec-7^pos^ and Siglec-9^pos^ NK cells (125). Siglec-10, another member of the Siglec family expressed by NK cells, is associated with decreased survival and impaired NK cell function in hepatocellular carcinoma (129). Therefore, targeting Siglec molecules on NK cells, or their ligands on malignant cells, might prove an attractive immunotherapeutic strategy to augment NK cell antitumor immunity (130). Supporting this hypothesis, a Siglec-7^neg^ NK-92 cell line exhibited high cytotoxicity against leukemia cells *in vitro* (131).

Overall, interactions between ligands and major activating receptors on NK cells are impaired in both HIV and cancer, with some common underlying mechanisms such as cleavage of membrane-bound receptor molecules by zinc-dependent endopeptidases such as MMPs and ADAMs. Therefore, drugs that prevent shedding of NK cell-activating ligands or receptors may enhance protection against development of cancer in PLWH. Several inhibitors of the metalloproteinase ADAM17, for instance, have already entered clinical trials and are being tested in combination with other therapeutics against cancer (132). Metalloproteinase inhibitors would also prevent CD16 shedding form the surface of NK cells, a mechanism that naturally occurs following CD16 ligation (133, 134) yet is dysregulated in HIV infection and cancer, thereby decreasing ADCC activity and cytotoxicity against HIV-infected or tumor cells. Moreover, increased levels of inhibitory receptors such as inhibitory KIRs or TIGIT on NK cells further contribute to decreased NK cell functions in PLWH (135, 136). Finally, unresolved inflammation is a hallmark of chronic HIV infection and is widely accepted to elicit malignant transformation of cells and carcinogenesis (137, 138). Several inflammatory mediators, such as TNF-α, IL-6, tumor-derived transforming growth factor β (TGF-β), and IL-10 have been shown to play a role in carcinogenesis. For instance, TGF-β is a cytokine endowed with immune-suppressing and anti-inflammatory properties that plays a key role in promoting NK cell dysfunction and is found elevated in both the tumor microenvironement and plasma of PLWH. In addition, TGF-β has been shown to elicit production of vascular endothelial growth factor by NK cells, thereby promoting tumor growth along with other cytokines chronically found elevated in PLWH (137, 139).

Altogether, these observations show that the ability of NK cells to eliminate tumor cells is impaired by the tumor microenvironment and further constrained in HIV infection, and that NK cell dysfunction in cART-treated PLWH may significantly contribute to their enhanced susceptibility to develop malignancies.

## Recent Advances in Development of NK Cell-Based Strategies for the Treatment of Cancer

NK cell-based immunotherapies rely on enhancement of endogenous NK cell activities in the tumor microenvironment or on adoptive transfer of NK cells with improved function. Strategies so far have included blockade of inhibitory NK cell receptors or immunosuppressive processes in the tumor microenvironment as well as enhancement of NK cell activation via cytokine stimulation or chimeric receptor expression (140). In this section, we will focus on strategies that could be of particular benefit in PLWH for elimination of HIV as well as HIV-associated cancers (Figure 1, right panel).

### mAb-Mediated Release of NK Cell Inhibition

Autologous NK cells are oftentimes suppressed by self HLA class-I molecules expressed on tumor cells that bind to inhibitory CD94/NKG2A or KIR. This can be circumvented by adoptive therapy of allogeneic NK cells with a KIR-HLA class I mismatch. Alternatively, release of inhibitory signals using mAbs that target HLA class I-binding NK cell inhibitory receptors represent another strategy to enhance NK cell antitumor functions. This approach might be particularly beneficial in PLWH, as HIV infection results in downmodulation of the major activating NK cell receptors.

NKG2A is a c-type lectin that has been shown to mediate NK cell suppression in both HIV infection and cancer. In particular, elevated expression of HLA-A has been linked to poor control of HIV (141). This deleterious effect is mediated by NKG2A^pos^ NK cells that are functionally suppressed by increased levels of HLA-E; whose expression is directly regulated by the availability of HLA class I-derived peptides. Whether increased HLA-A also correlates with poor outcome in cancer remains to be determined. Overexpression of HLA-E by tumor cells has long been proposed as a mechanism of escape from the action of NK cells (142). For instance, enhanced expression of HLA-E in hepatocarcinomas is driven by IL-10 released in the tumor micro-environment and is associated with enhanced NKG2A expression, a profile that correlates with NK cell exhaustion/anergy, as measured by low IFN-γ intracellular production upon stimulation with IL-12, and with a poorer prognosis (143). Failure to achieve remission in AML patients has been linked to impaired function of NK cells that upregulated NKG2A (144), and expression of HLA-E in multiple myeloma cells decreases NK cell cytotoxicity (145). Accordingly, efficacy of a specific IgG4 mAb that targets NKG2A (Monalizumab) is currently being assessed in various tumor settings along with other mAbs (61). Promising results were obtained in phase II trials in combination with the anti-EGFR antibody Cetuximab in head and neck cancers (146). Interestingly, Monalizumab targets both T cell and NK cell responses, promoting effector T cell responses in combination with anti-PDL1 and enhancing NK cell effector functions, including ADCC. Whether therapeutic blockade of HLA-E:NKG2A interaction, potentially in combination with PD-1 signaling blockade, could significantly improve control of HIV remains to be evaluated. NKG2A also significantly contributes to NK cell education in the early stages of NK cell ontogenesis. Accordingly, administration of Monalizumab has been suggested to promote NK cell alloreactivity against malignant cells when administered early after haplo-HSCT, thereby circumventing the need for a KIR-mismatched donor (147).

Inhibitory KIRs represent another interesting target for immunotherapies. For instance, Lirilumab, an IgG4 mAb that targets KIR2DL1/2/3 and KIR2DS1/2 has been evaluated in several clinical trials in combination with different mAbs in AML (phase II NCT02399917), MDS (phase II NCT02599649), lymphoma (phase II NCT01592370), and CLL (phase I NCT02481297). However, long-term use of inhibitory KIR blocking agents might lead to desensitization of NK cells (60). Finally, the recent discovery of HLA-F OCs' ability to bind KIRs, and particularly KIR3DS1 that has a widespread influence in human diseases including HIV, has made HLA-F a target of significant interest for therapies to enhance anti-tumor function of NK cells that might be particularly relevant for PLWH with malignancies.

Numerous antibody-based immune checkpoint inhibitors currently under investigation target the interaction of PD-1 or CTLA-4 and their cognate ligands on tumor cells, in order to boost the power of tumor-specific CD8^pos^ T cells. In particular, clinical studies assessing the blockade of PD-1 or its ligand PD-L1 reported potent therapeutic efficacy against several cancers such as melanoma and non-small cell lung cancer. Selective PD-1 expression on CD56^dim^CD57^pos^ mature NK cells in some but not all healthy individuals has been reported (148) and associated with functional defects (149). However, overall expression and functional relevance of those markers on NK cells in health and disease is still unclear, and recent studies suggest that blockade of CTLA-4 and PD-1 might enhance NK cell anti-tumor activity mostly via indirect mechanisms (150). Interestingly, PD-1 also mediates T-cell exhaustion in chronic HIV infection, and dual immune checkpoint blockade targeting PD-1 and IL-10 significantly enhances NK cell function through reversal of adaptive immune exhaustion in PLWH (151). Therefore, immunotherapeutic interventions targeting PD-1 may augment NK cell responses against both HIV and tumors in PLWH.

Another immune checkpoint inhibitor currently tested in clinic is a mAb targeting Tim-3, a receptor associated with exhaustion in T cells. Tim-3 has been proposed to mark mature NK cells, with chronic Tim-3 upregulation being associated with NK cell dysfunction, yet the precise impact of TIM-3 expression on NK cell function require further investigations (152). While Tim-3 has been shown to be upregulated on NK cells in various tumors, studies dissecting the effects of Tim-3 blockade on NK cell function in cancer settings have yielded mixed results (150).

Finally, even though it represents a promising approach for the treatment of cancer, administration of mAbs targeting regulatory immune checkpoint molecules has been associated with toxicities known as immune-related adverse events (irAEs) (153). irAEs are mainly caused by the release of inhibitory mechanisms that normally constrain the immune response, leading to various local and systemic autoimmune responses. Clinical benefit of immune checkpoint therapy is also restricted to a subset of patients. Several mechanisms of resistance to immune checkpoint inhibition have been described (154, 155). Notably, cancer therapy based on administration of mAbs promotes the induction of antibodies against such humanized mAbs and it is not clear yet whether such antibodies do or do not play a role by neutralizing the effects of the therapy. However, the potential of such antibodies to induce hypersensitivity reactions need to be considered.

### mAb-Based NK Cell Activation

Antibody therapy that targets activating NK cell receptors is another strategy that has shown efficacy in certain malignancies. Elotuzumab, an antibody that targets SLAMF7, directly activates NK cells and can simultaneously induce ADCC by coating multiple myeloma cells, which express SLAMF7. The ability of a therapeutic mAb to induce ADCC results in potent NK cell activation and led to the design of bi-specific and tri-specific killer cell engagers, BiKEs and TriKEs, respectively. These single-chain variable fragment recombinant reagents can bind the tumor cells and NK cells via CD16 to induce direct killing via ADCC. This technique has been used in clinical trials where Hodgkin target cells expressing CD30 were linked to CD16 expressed on NK cells (156). The anti-CD16XCD33 BiKE activation can override the inhibitory signals mediated by ligation of inhibitory NK cell receptors and their HLA class I ligands expressed on AML (157) and MDS (158) targets. However, BiKEs do not promote *in vivo* proliferation and survival of NK cells. To overcome this issue, TriKEs were manufactured to engage the IL-15 receptor and are evaluated in different tumor pathologies (159–161). Use of therapeutic mAbs with potent ADCC activity may lead to substantial benefit in PLWH who present high frequencies of NK cells with enhanced antibody-dependent activation, as described in the last section.

### Activation of NK Cells via CAR

A new tool for immunotherapy is chimeric antigen receptor (CAR)-engineered NK cells. CAR are artificial receptors composed of an extracellular antibody-derived tumor antigen binding domain as well as transmembrane and intracellular domains for activating signal transduction (162). Thus far, CAR T cells have been developed and successfully employed in the treatment of hematological malignancies. However, use of CAR-T cells has been limited as therapy for solid tumors and triggered numerous severe side effects in clinical trials that can be overcome with the use of CAR-NK cells. These include graft-vs.-host disease, cytokine release syndrome, and off-target toxicities. Moreover, CAR-NK cells can also eliminate tumor cells in a CAR-independent manner through recognition of ligands expressed on tumor cells by a range of activating receptors such as NKp30, NKG2D, DNAM-I, providing another advantage to use CAR-NK cells over CAR-T cells for cancer immunotherapies (163, 164). However, safety and efficacy of CAR-NK cells in humans need to be fully evaluated as only few clinical trials have been using CAR-NK cells up to now. One issue pertaining to CAR-NK cells is their limited *in vivo* persistence. To circumvent this restriction, a phase II trial is currently assessing the persistence and anti-tumor activity of IL-15- and caspase-9 suicide gene-transduced CD28-CAR-NK cells in B cell lymphoma (NCT03056339). Alternatively, CAR expression in adaptive NK cell subsets discussed in the next section may overcome expansion and persistence issues while simultaneously boosting anti-tumor activity. Finally, implementation of CAR-based strategies optimized for NK cells is warranted. For instance, induced pluripotent stem cell (iPSC)-derived NK cells transduced with novel CAR constructs that include NK cell-specific signaling domains instead of CD3ζ signaling-based domains are being evaluated and may significantly enhance their potency (165). Importantly, while CAR-T cell-based clinical trials have failed to provide clinical benefit and HIV viral suppression in PLWH, advanced CAR strategies that are developed specifically for NK cells in the cancer field can benefit PLWH as they could be applied to efficiently redirect NK cell functions toward HIV-infected cells (166).

### Immunotherapeutic Potential of Adaptive NK Cells

While NK cells are classically viewed as non-specific effector cells of the innate immune system, a vast amount of independent studies has demonstrated that subsets of murine, non-human primate and human NK cells are capable of adaptive immune functions, including antigen-dependent expansion and long-lived immunological memory (167, 168). Adaptive NK cell-based immunotherapies may circumvent many of the limitations inherent to the various strategies tested thus far to harness anti-tumor functions of conventional NK cells.

The best characterized adaptive NK cell subset in humans is the one driven by HCMV infection, originally identified as a population of NK cells expressing high levels of the activating CD94/NKG2C receptor and the marker of terminal differentiation CD57, which expand upon HCMV infection or reactivation and can persist for years at high frequency in HCMV-seropositive individuals (169–173). Corroborating the adaptive features of this NK cell subset, it was recently shown that expansion and differentiation of this CD94/NKG2C^pos^ NK cell subset is driven by the HCMV UL40 peptide presented by HLA-E, the ligand for NKG2C (174).

The CD94/NKG2C^pos^ NK cell population largely overlaps with an FcεRIγ adaptor protein-deficient memory NK cell subset with enhanced antibody-dependent functions (FcγRΔg NK cells) that has more recently also been characterized in HCMV-seropositive subjects (175–184) and rhCMV-positive macaques (185). Adaptive characteristics of FcγRΔg NK cells include a distinctive epigenetic signature close to that of memory CD8^pos^ T cells, endowing these adaptive NK cells with specialized functions such as enhanced responses to CD16 cross-linking, potent IFN-γ production to selective stimuli and reduced activation by innate cytokines.

Interestingly, adaptive CD94/NKG2C^pos^ NK cells proliferate not only in response to CMV reactivation or infection in patients receiving hematopoietic transplantation (169, 172, 186–188), but also upon *de novo* infection with different viruses including HIV and upon HCMV reactivation in PLWH (171, 189, 190). Several reports strongly suggest that HCMV-associated adaptive NK cells improve control of HIV infection. Higher frequencies of CD94/NKG2C^pos^ NK cells during primary HIV infection are linked to lower viral set points, are predictive of higher CD4^pos^ T cell counts and of an overall better outcome in treated PLWH (191, 192). In contrast, individuals with NKG2C gene deletions are more susceptible to HIV infection and once infected may have accelerated disease progression (193). Finally, in HCMV-seropositive PLWH, CD94/NKG2C^pos^ NK cells exhibiting adaptive signatures of FcγRΔg NK cells present conserved effector functions (190). The beneficial effect of adaptive CD94/NKG2C^pos^ NK cells has also been demonstrated in cancer settings. HCMV reactivation has been linked to longer relapse-free survival in patients with hematological malignancies receiving allogeneic hematopoietic cell transplantation (194). More specifically, expansion of adaptive NKG2C^pos^CD57^pos^ NK cells upon HCMV reactivation after HCT is associated with reduced leukemia relapse (195, 196). Of note, specific phenotypic signatures have been associated with this NK cell adaptive subset and include lack of NKG2A expression. As a result, these cells are intrinsically insensitive to tumor-mediated suppression through HLA-E. Therefore, HCMV-associated adaptive NK cells represent an attractive subset of NK cells that could be exploited instead of conventional NK cells to limit cancer incidence in PLWH, particularly in combination with tumor-targeting therapeutic antibodies that efficiently promote NK cell-mediated ADCC.

NK cell memory has been described against multiple viral, bacterial, and tumor antigens, and can also be induced by brief exposure to specific cytokines. Indeed, NK cells can differentiate into cytokine-induced memory-like (CIML) NK cells that display enhanced effector functions after a short pre-activation with a combination of IL-12, IL-15, and IL-18 followed by a prolonged rest period (197). Re-stimulation of CIML NK cells using leukemia target cells, cytokines or FcγRIIIa ligation is associated with increased responsiveness that can be retained for several weeks following their initial pre-activation (197–202). CD56^bright^ and CD56^dim^ NK cells both have the potential to differentiate into CIML NK cells (197). Potent effector functions of CIML NK cells have been linked to expression of the high-affinity IL-2 receptor α*βγ* (IL-2Rα*βγ*), demethylation of the conserved upstream non-coding enhancer region of the IFN-γ gene, recruitment of anergic unlicensed NK cells, enhanced antibody-mediated functions and release from KIR-mediated inhibition (198, 200, 201, 203). Therefore, superior functionality of CIML NK cells is not affected by prior licensing through HLA class-I molecules. Compared to control NK cells, CIML NK cells have been shown to express higher levels of CD56, CD94, NKG2A, NKG2D, NKp46, CD25, NKp30, NKp44, CD62L, CD27, TRAIL, perforin and granzyme B, and lower levels of CD16, whereas NKG2C expression was found similar between control and CIML NK cells (197, 199).

The long-lived properties of CIML NK cells have tremendous potential to be exploited for cancer immunotherapy, and preliminary results from a first-in-human phase 1 clinical trial have shown that NK cells pre-activated with IL-12, IL-15, and IL-18 can expand *in vivo* and exert robust responses against leukemia targets, leading to remission in a subset of AML patients (199). A better understanding of the mechanisms behind CIML NK cell responses may lead to novel strategies to further enhance their antitumor function. For instance, recent studies suggested that targeting the interaction between SEMA7A, a potent immunomodulator expressed by cytokine-activated NK cells, and integrin-β1 might provide a novel immunotherapeutic approach to potentiate antitumor activity of CIML NK cells (204).

Strikingly, burgeoning evidence also supports the existence of true antigen-specific memory NK cells in humans (174, 177, 205), including a recent report of human HIV-specific memory NK cells (168). While further studies are warranted to fully characterize human antigen-specific NK cells and define the mechanisms underlying NK cell memory formation and maintenance, it is possible that adaptive NK cells that can specifically recognize tumor-associated antigens and efficiently eliminate cancerous cells develop in cancer patients. Vaccines including components to boost tumor-specific NK cells or infusion of expanded tumor-specific NK cells represent attractive avenues for the development of novel therapeutic interventions.

Overall, the immunotherapeutic potential of adaptive NK cells is expected to exceed that of conventional NK cells as they may overcome some of the major limitations faced in NK cell-based cancer therapies that have been evaluated so far in pre-clinical or clinical studies. For instance, adaptive NK cells can be expanded *ex vivo*, are long-lived and persist *in vivo*, are less sensitive to regulatory T cells-mediated suppression (206) or myeloid-derived suppressor cell inhibition (207) and can achieve significantly enhanced antibody-dependent functions (194) or antigen-specific cytotoxicity (168). Importantly, HCMV-dependent adaptive NK cells are increased 7-fold (181) and confer protection in PLWH (191, 192). Therefore, exploitation of adaptive NK cells may represent an attractive strategy to efficiently prevent or treat malignancies in PLWH.

## Author Contributions

OL and SJ contributed to writing of specific sections. RR and SJ edited the final version of the manuscript.

### Conflict of Interest Statement

The authors declare that the research was conducted in the absence of any commercial or financial relationships that could be construed as a potential conflict of interest.
